# Supplementary material for: RNA Sequencing Reveals Inflammatory and Metabolic Changes in the Lung and Brain After Carbon Black and Naphthalene Whole Body Inhalation Exposure in a Rodent Model of Military Burn Pit Exposures
Source: Int J Mol Sci. 2025 Jul 26;26(15):7238. doi: 10.3390/ijms26157238 (PMC12346809; doi:10.3390/ijms26157238)
Supplement: Supplementary file 1 [file ijms-26-07238-s001.zip › ijms-3722693-supplementary.pdf]

Statistical model for selecting a predictive gene signature of CBN exposure

We sought to identify a set of genes whose expression levels have predictive value for differentiating between sham air and CBN-exposed tissue samples. These genes could potentially be used as biomarkers to predict harmful effects of toxicant exposure, but additional experiments are necessary to confirm their predictive value beyond the scope of this experiment.

Using normalized, log2 transformed counts per million (cpm) values of differentially expressed genes from lung tissue RNA-seq data (278 genes, FDR p-value ≤ 0.05), we selected predictive variables using an elastic net penalized regression model with the R package glmnet [181] with the default penalty parameter. Penalized regression selects a smaller, more meaningful subset of predictor variables in the model by assigning a penalty that decreases as the model includes fewer coefficients. We tested other penalized regression models, LASSO [174] with glmnet and SCAD [172] with the ncvreg [171] R package. The results were qualitatively similar with LASSO being the most conservative, as it generally selects one variable from a group of correlated variables. In contrast, elastic net select a cluster of correlated variables. We chose elastic net for the final model, which retained 100 genes, because it was designed specifically for prediction and variable selection with highly correlated gene expression data [182].

We validated the model using Leave-One-Out Cross-Validation (LOO-CV)) [176]. For each sample S_j, we created a training set of all data from that tissue type, excluding S_j. Then we ran an elastic net regression model on that training set to predict treatment status (sham vs CBN). Finally, we used this model to estimate the probability that the S_j

was exposed to CBN. This probability should be small for samples exposed to sham air, and large for samples exposed to CBN. This is what we observed, supporting that the elastic net model has predictive validity for this experiment, and is not just fitting noise.

It is also important to provide some intuition on why using a predictive or classification model adds value to the differential expression analysis. As an example, consider the gene Noxo1. The expression values in log2 cpm are as follows; CBN: 4.11, 4.22, 4.0, 4.42, 4.06; Sham: 2.05, 2.81, 3.1, 2.45, 2.68. The expression values in the CBN condition are higher than in the Sham condition. There is also a large margin between the values – the largest gene expression value in the Sham condition is 3.1 and the smallest expression value in the CBN condition is 4.0.

Given this, how could we predict the exposure condition, using the expression values? If we were told that the expression was 2.6 log2 cpm, we would likely predict that the sample had been in the Sham condition. But what if we were told that the expression was 3.7 log2 cpm? In principle, we could draw a line at 3.55 log cpm, and categorize any sample with Noxo1 log2 cpm expression greater than 3.55 as belonging the CBN exposure group. We could do the opposite with an expression value less than 3.55.

While reasonable, there are two problems with this approach. First, there is no objective way to pick the threshold. There are infinitely many thresholds between 3.1 and 4.0 that would classify the existing data perfectly. Second, the threshold approach does not say anything about the probability that a sample belongs to an exposure group. Even if we picked 3.55 as the threshold, there is a difference between expression values of 3.6 and 3.95. In the former case, we should be more uncertain about the exposure group. In the latter case, we should be more certain that the sample was exposed to CBN. Therefore,

it would help to have an approach that 1) can be validated and 2) measures the probability of belonging to each group.

There are many algorithms in statistics and machine learning that try to solve this problem. Elastic net finds a linear function of expression values that classifies the data and estimates the probability that a sample was exposed to CBN. It is important to work with standardized expression values when using elastic net. For example, the overall mean expression for Noxo1 was 3.39 and the standard deviation was 0.86 log cpm. If the measured expression for Noxo1 was 3.7 log cpm, then the standardized expression is approximately $(3.7 - 3.39)/0.86 \cong 0.36$, i.e., the expression is 0.36 standard deviations above the mean. Using non-standardized expression values will lead to unstable and inaccurate predictions with elastic net.

The classifier function C is $C = A \times (\sum_{Gene\ i} Sign_i \times Weight_i \times Std.Exp_i)$. For each gene, we have the standardized expression, its weight in the model and the sign of the weight. If higher expression values predict CBN exposure, the gene will have positive sign (e.g. *Kcnq3*), otherwise, it will have a negative sign (e.g. *Sirpb3*). The weights sum to 100% across all genes. There are 181 genes with zero weight in the model – the model did not select them. Of the remaining 97, the top ten account for 27% of the total weight. The largest weight is for ENSRNOG00000068035 (3.6%). Thus, the elastic net spreads the responsibility for the prediction across many genes. The final term, A, is a constant that reflects the amount of signal in the data.

Given a classifier value $C = c$, elastic net predicts that the sample was exposed to CBN with probability p, where $p = \exp(c)/(1 + \exp(c))$, which is the inverse logit function. The estimated probabilities, $\hat{p}$, are used to validate the model. For samples that were

exposed to CBN, $\hat{p}$ should be close to 1. For samples that had the Sham exposure, $\hat{p}$ should be close to zero. We use cross-validation, as described above, to avoid overfitting. Internally, elastic net chooses the number of selected genes and the weights of each gene to find a good balance between fitting the data and model complexity.

Finally, we want to explain why some genes had higher weights in the model than others. Elastic net is an extension of linear or logistic regression to the situation where the number of variables, p (genes), is larger than the number of cases, n (rats). In linear regression, we can use an added-variables plot to understand why some variables have more weight in the model than others. Suppose we have a response Y, a variable of interest X and a set of confounders Z. Usually in practice (not necessarily in theory), there are two requirements for X to be important in the regression model. First, X should have a strong correlation with Y. Second, X should be hard to predict from Z alone. An added-variables plot shows the correlation between X and Y, after removing the contribution of Z from both variables. To our knowledge, we cannot apply an added-variables plot to this data. The algorithm does not work because $p \gg n$, and because we have a binary response variable (CBN vs Sham).

In our plot of modeling outcomes, the genes with the highest weights have higher values on the y-axis and lower values on the x-axis. Elastic net is searching for genes that are predictive of condition by themselves (y-axis) and whose expression is hard to predict from the other genes (to the left on the x-axis). The values on the y-axis are the absolute standardized differences between conditions. The values on the x-axis are defined as the correlation between a gene's expression and its predicted expression, again using LOO-CV. This time, we used ridge regression to get the predicted gene's expression values,

using the other 277 genes in the dataset. The higher this correlation, the more explainable the expression is according to the other genes' expression. Therefore, for two genes with an equal standardized expression difference between conditions, the one that is less predictable will have a higher weight. The contribution from both axes is indicated by the points that are redder: the color for gene i is the absolute value of $(A \times Sign_i \times Weight_i)$. It is important to emphasize that all genes in the model contribute to the prediction as a team. We have highlighted the top ten contributors to the model to demonstrate its biological plausibility. Even though elastic net can be fit with $n = 10$ cases, we should be very cautious about extrapolating these results to other contexts.
